# Huntington's Disease: The Most Curable Incurable Brain Disorder?

**DOI:** 10.1016/j.ebiom.2016.05.023

**Published:** 2016-05-19

**Authors:** Edward J. Wild

**Affiliations:** Huntington's Disease Centre, UCL Institute of Neurology, National Hospital for Neurology & Neurosurgery, Queen Square, London, UK

Many scientists and people from families affected by Huntington's disease prefer to avoid ‘the C-word’: cure. Acknowledging this, I still feel it is justifiable to describe Huntington's as the most curable incurable brain disorder.

Everyone with HD has the same basic problem – a CAG expansion mutation in *HTT*, encoding a polyglutamine tract in the huntingtin protein (The Huntington's Disease Collaborative Research Group, 1993). Everyone with that problem will develop HD at some point, slowly succumbing to progressive cognitive, motor and psychiatric impairment. It is almost fully penetrant and truly dominant, and the genetic test is bleakly reliable.

The more prevalent neurodegenerative diseases like Alzheimer's and Parkinson's lack this certainty: only a tiny minority of cases have a defined genetic cause. Therapeutic targets must be prosecuted on the balance of probability. In HD, we operate ‘beyond reasonable doubt’: mutant huntingtin is a smoking gun. Anything that cannot be connected to the mutation or protein may confidently be discarded.

History has shown HD is an easy problem to define, for which it is surprisingly difficult to find solutions. Twenty-two years of research since the gene discovery in 1993 (see Fig. 1) has revealed much about huntingtin protein and its toxic twin, resulting in numerous genetically-accurate cell and animal models and theories of molecular and cellular pathogenesis – yielding multiple therapeutic targets (Ross et al., 2014, Wild and Tabrizi, 2014).

Based on our early understanding of its pathogenesis, the HD field tested an array of already-licensed neuroactive drugs (including baclofen, lamotrigine, riluzole and minocycline), plus a whole shelf of ‘nutraceuticals’ held to be generally good for neurons (creatine, coenzyme Q, fish oil and others). Nothing worked (Mestre et al., 2009).

A failed drug does not imply a failed trial. Collectively, these experiences improved the field's ability to design and run trials that can give a definitive answer, and to prioritise therapeutic targets and generate comprehensive preclinical data to inform decisions about advancing to clinical trials.

We also have at our disposal a new generation of drugs that take aim at the known cause of HD. 2005 saw the first attempt to ‘silence’ the *HTT* gene in an HD mouse model, using RNA interference. Originally discovered in petunia flowers, and eventually found to be a natural means of regulating post-transcriptional gene expression across species (Matzke and Matzke, 2004), ‘gene silencing’ as a therapeutic approach involves designing and synthesising an oligonucleotide molecule with a sequence complementary to the messenger RNA of the gene of interest. mRNA bound to the drug molecule is degraded by cellular enzymes, diminishing the manufacture of the target protein. Conceptually, the technique is as simple as turning off the water in an overflowing bathtub; but that does not mean it is easy to implement for a neurodegenerative disease – even one with a clear, dominant genetic cause.

The mice whose brains were injected with the RNA interference compound did not just deteriorate more slowly – their motor problems improved, and regression of neuropathology was also seen (Harper et al., 2005). Similar improvements have been seen now with several such drugs in different model animals. These models may be imperfect but are the only current means by which preclinical efficacy can be judged.

Meanwhile, a quiet revolution has been taking place in the field of HD biomarkers. The need for measures that can give an early, objective indication of progression or therapeutic effect is common to all neurodegenerative diseases. In HD, we can identify people destined to get the disease, but a major challenge is measuring whether a drug is working to prevent onset. By any established clinical measure, mutation carriers are indistinguishable from controls until they develop symptoms.

So, large cohorts of patients and mutation carriers were assembled and studied over years, to determine what measurements were most reliable for predicting onset and progression. The result was a toolkit of imaging, clinical and cognitive biomarkers that can be used to facilitate clinical trials (Tabrizi et al., 2013). Last year, we reported the first quantification of mutant huntingtin protein in cerebrospinal fluid (CSF), and showed that its concentration predicts clinical features of HD. This is the smoking gun itself, released from the neurons it is killing (Wild et al., 2015). We now need to enlist large cohorts of well-characterised HD mutation carriers and study their CSF comprehensively: this is the aim of our nascent HDClarity study (http://hdclarity.net).

In September 2015, the first dose of an antisense oligonucleotide drug – a chemically-modified single DNA strand – was injected into the CSF of a patient with HD (BBC News, 2015). The global trial, led by our centre at UCL, is designed to test the safety of the drug, IONIS-HTT_Rx_, developed by Ionis Pharmaceuticals, aimed at suppressing production of huntingtin in the human brain (ClinicalTrials.gov, 2015). Among other measures, huntingtin will be quantified in CSF to look for evidence that the drug is engaging with its target.

This trial marks a huge step towards treatments to improve the situation of HD-affected families. It owes its existence to decades in parallel pursuit of basic and clinical pathobiology, therapeutic development, biomarker discovery, clinical trials and patient education (e.g. http://hdbuzz.net). Testing the efficacy of this first huntingtin-lowering drug alone will take several years, and of course there may be setbacks ahead. It is to be hoped that whatever can be accomplished in HD will illuminate the global fight against neurodegenerative disease.

## Figures and Tables

**Fig. 1:**
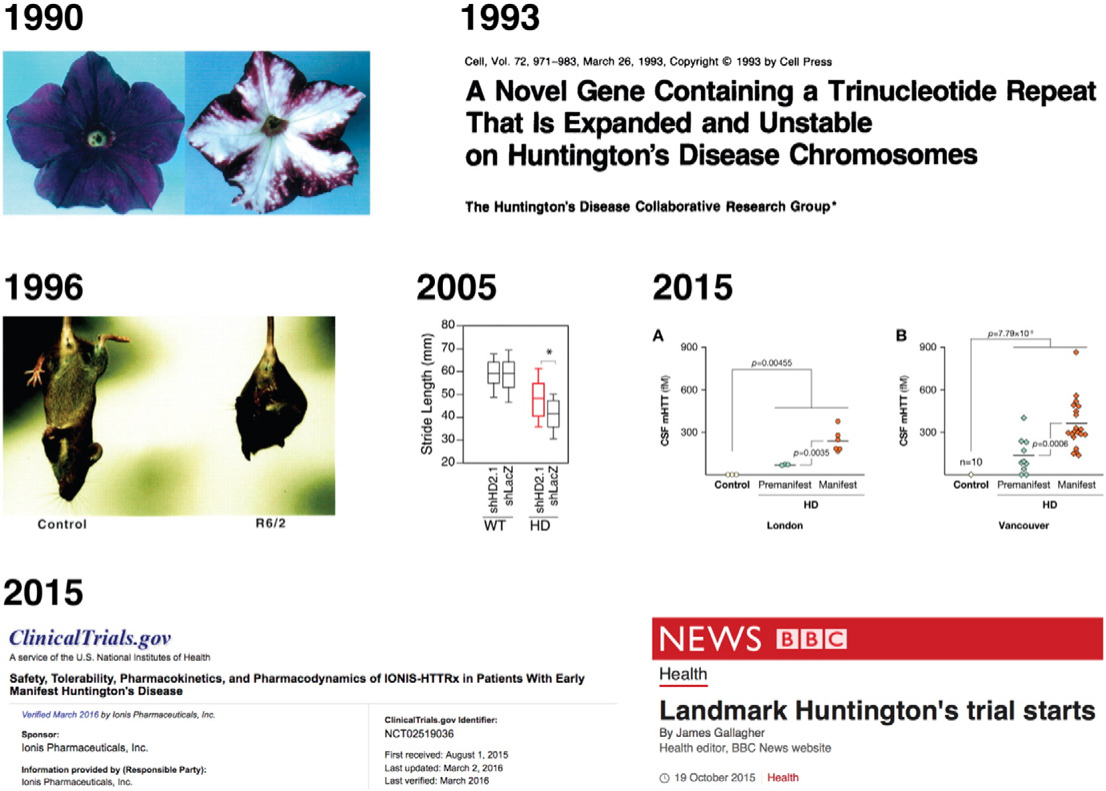
Some milestones on the road to huntingtin-lowering investigational therapies for HD patients. 1990, RNA interference is described after overexpression of pigmentation enzymes in petunia flowers (left) is seen to produce paradoxical reductions in pigment (right) due to co-suppression by interfering RNA. Modified from Matzke and Matzke, 2004. 1993, the genetic expansion in HTT responsible for HD is identified by a global collaborative research group (reproduced from The Huntington's Disease Collaborative Research Group, 1993 with permission). 1996, Bates and colleagues report the first transgenic mouse model of HD, the R6/2, still widely in use (reproduced from Mangiarini et al., 1996 with permission). 2005, the first report of oligonucleotide-based huntingtin suppression in a mouse model of HD resulting in improved motor function towards wild-type level. Red box shows transgenic animals treated with active compound. (modified from Harper et al., 2005.) 2015 (middle row), first successful quantification of mutant huntingtin protein in cerebrospinal fluid from HD mutation carriers using a novel single-molecule counting immunoassay (modified from Wild et al., 2015 with permission.) 2015 (bottom row), the first trial of an oligonucleotide huntingtin-lowering compound, IONIS-HTTRx, begins in HD patients (screenshots from ClinicalTrials.gov and BBC News, 2015, edited for clarity).
